# Large primary cardiac tumor penetrating the right ventricle: 3-dimensional printing-based surgical planning

**DOI:** 10.1016/j.xjtc.2021.10.061

**Published:** 2021-11-09

**Authors:** Eunji Kim, Won Kyung Pyo, Dong Hyun Yang, Joon Bum Kim

**Affiliations:** a Department of Thoracic and Cardiovascular Surgery, Asan Medical Center, University of Ulsan College of Medicine, Seoul, South Korea; b Department of Radiology, Asan Medical Center, University of Ulsan College of Medicine, Seoul, South Korea

**Figure:**
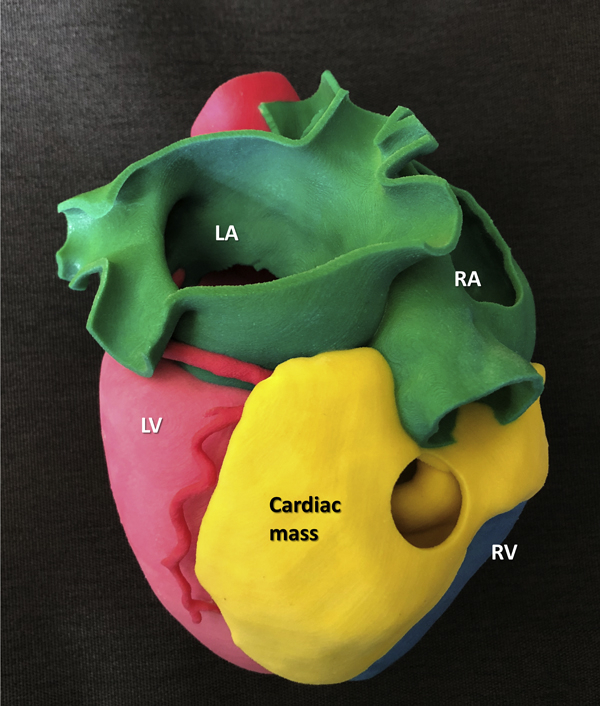
A 3-dimensional printing heart model viewed from inferoposterior aspect.

**Central Message**

A 3-dimensional printing model may be an important guiding tool for establishing surgical planning in complex cardiac tumors.

See Commentaries on pages 41 and 43.

The primary cardiac tumor is extremely rare, constituting 0.3% to 0.7% of all cardiac tumors.^1^ Although complete resection is the mainstay in the management of cardiac tumors, it is technically challenging because of their extensiveness or involvement in vital structures. Preoperative imaging examinations including echocardiography and computed tomography scan provide information regarding the anatomical relationship between cardiac tumor and adjacent structures; however, it might be insufficient to determine operability.^2^ We report a case of a large primary cardiac tumor penetrating the right ventricle in which surgical planning was established based on 3-dimensional (3D) printing model.

## Case Report

**Figure 1:**
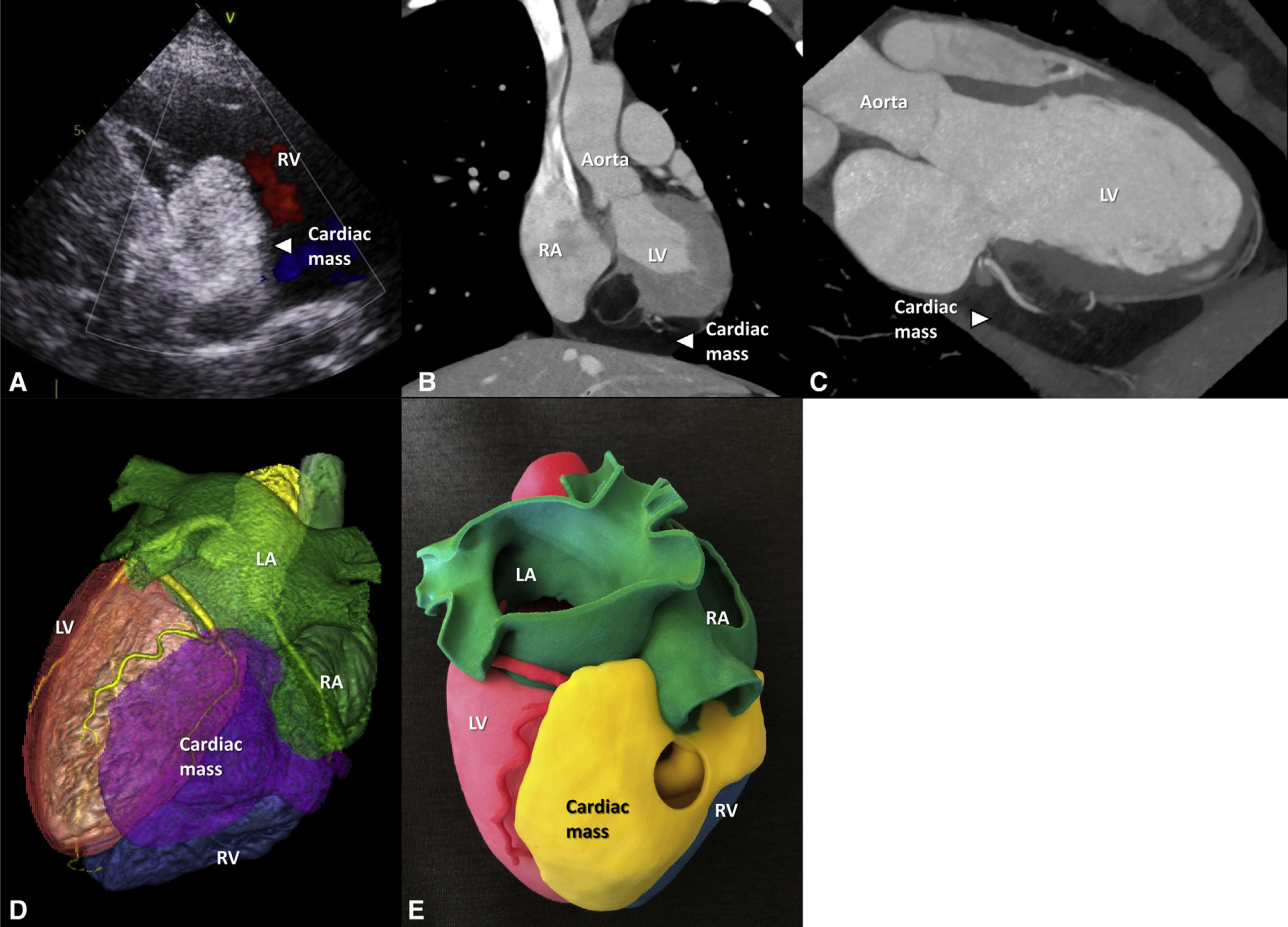
Preoperative image findings of cardiac tumor. A, Transthoracic echocardiographic image. B-D, Computed tomography image of the heart showing a cardiac mass measuring 66 mm × 70 mm × 40 mm. E, Three-dimensional printing model. *RV*, Right ventricle; *RA*, right atrium; *LV*, left ventricle; *LA*, left atrium.

A 33-year-old female patient was referred to our institution due to an incidentally found cardiac tumor. On transthoracic echocardiography, an echogenic mass at the inferoposterior right ventricular (RV) wall extending to the mitral annulus level was noted without deterioration of valvular or ventricular function (Figure 1, *A*). Computed tomography of the heart revealed a cardiac mass (66 mm × 70 mm × 40 mm) that, located at the inferior epicardial space, was penetrating and protruding into the RV chamber (Figure 1, *B-D*). Radiologic findings suggested a liposarcoma. For further understanding of spatial orientation and relation to adjacent structures, a 3D printing model was constructed using a gypsum-like cast model (Projet 460 printer and VisiJet PXL Core powder, VisiJet PXL clear binder and color bonds; 3D Systems, Rock Hill, SC) and resectability of the tumor was determined (Figure 1, *E*).^3^ We planned RV mass removal with the aim of making a confirmative diagnosis and achieving curative resection.

**Figure 2:**
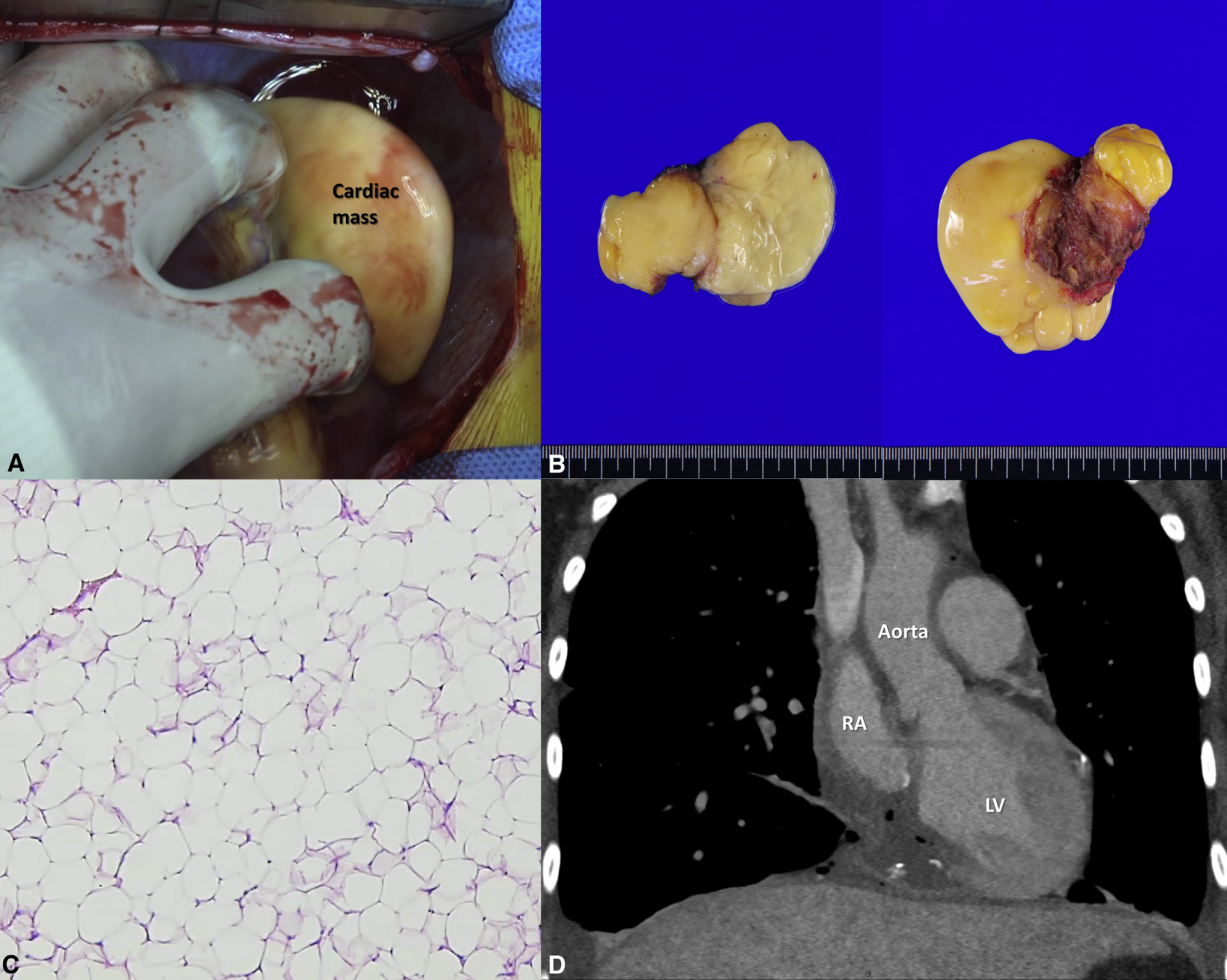
A, Cardiac tumor observed in the operative field. B, Gross appearance. C, Histopathologic findings of the cardiac tumor. D, Postoperative computed tomography image of the heart. *RA*, Right atrium; *LV*, left ventricle.

After sternotomy, the base of the tumor was found at the inferior side over the right atrioventricular groove (Figure 2, *A*). Cardiopulmonary bypass was established via the ascending aorta, superior vena cava, and the right femoral vein. The right atrium was opened under a beating-heart state, and atrial-side tumor margin was demarcated. The aorta was crossclamped and then 1 L of del Nido solution was administered via an aortic root cannula.

The septoposterior commissure of tricuspid valve (TV) was completely divided to eliminate the tumor, and the posterior descending artery, which was entirely encircled by the tumor, was sacrificed. The inferior part of RV, atrial septum, and the coronary sinus orifice were defective after tumor resection. After direct closure of atrial septal defect, the RV wall was repaired by double-patch technique using bovine pericardial patch. The coronary sinus was recreated and then the right atrioventricular groove, right atrium, and inferior vena cava were reconstructed by single bovine pericardial patch. Gross structure of TV was restored after atrioventricular repair and a plication stitch was added to the septoposterior commissure (Video 1). The aortic crossclamping and cardiopulmonary bypass times were 77 and 90 minutes, respectively. Frozen section examination revealed all negative resection margins. Final pathologic examination verified low-grade lipogenic tumor with clear resection margin and negative result of immunohistochemistry staining indicated lower possibility of well-differentiated liposarcoma. (Figure 2, *B* and *C*). Postoperative computed tomography and echocardiography revealed no residual mass (Figure 2, *D*). The patient was discharged on postoperative day 4 without perioperative complications.

The study was approved by the institutional review board of Asan Medical Center (IRB approval no., 2021-0640), and the statement of patient informed consent was written by the patient.

## Comment

General principle in the treatment of primary cardiac tumor suggests that surgical resection should always be considered with priority to establish a confirmative diagnosis, offer chances of curative resection, and prevent later malignant transformation, unless there is a possibility of lymphoproliferative disorders where chemo-/radiotherapy are the first-line therapies.^4^ Incisional biopsy may trigger invasion to adjacent structures by disrupting the tumor and consequently reducing the chance of a disease cure; thus, complete resection should be the primary choice whenever possible.^5^ Referring to previous studies, out Heart Team decided to undergo complete resection after thorough discussion.

In the present case, the cardiac tumor was violating various structures including the RV, TV, and coronary artery branch, and 2-dimensional images provided insufficient information to surgeon to have comprehensive perceptions on anatomical relationship between the tumor and adjacent structures, and determine operability. The 3D-printing model enabled surgeons to identify the tumor extension precisely and establish surgical strategy by repeated hands-on simulations. Based on various scenarios prepared preoperatively, we could operate on this complex cardiac mass with confidence and accomplish successful surgery by achieving complete resection with negative resection margins. We speculate the surgery might have not been executed if it had been determined inoperable depending upon the ordinary decision-making process considering its extensiveness and complexity. In conclusion, a 3D printing model may be an important guiding tool for determining operability and establishing surgical planning in complex cardiac tumors.

## Supplementary Data

Video 1 The video presents a brief introduction of the patient including preoperative imaging evaluation of the cardiac tumor, surgical procedure, and postoperative course. Video available at: https://www.jtcvs.org/article/S2666-2507(21)00756-2/fulltext.
