# Supplementary figures and images for: Large primary cardiac tumor penetrating the right ventricle: 3-dimensional printing-based surgical planning
Source: JTCVS Tech. 2021 Nov 9;11:37–40. doi: 10.1016/j.xjtc.2021.10.061 (PMC8828928; doi:10.1016/j.xjtc.2021.10.061)

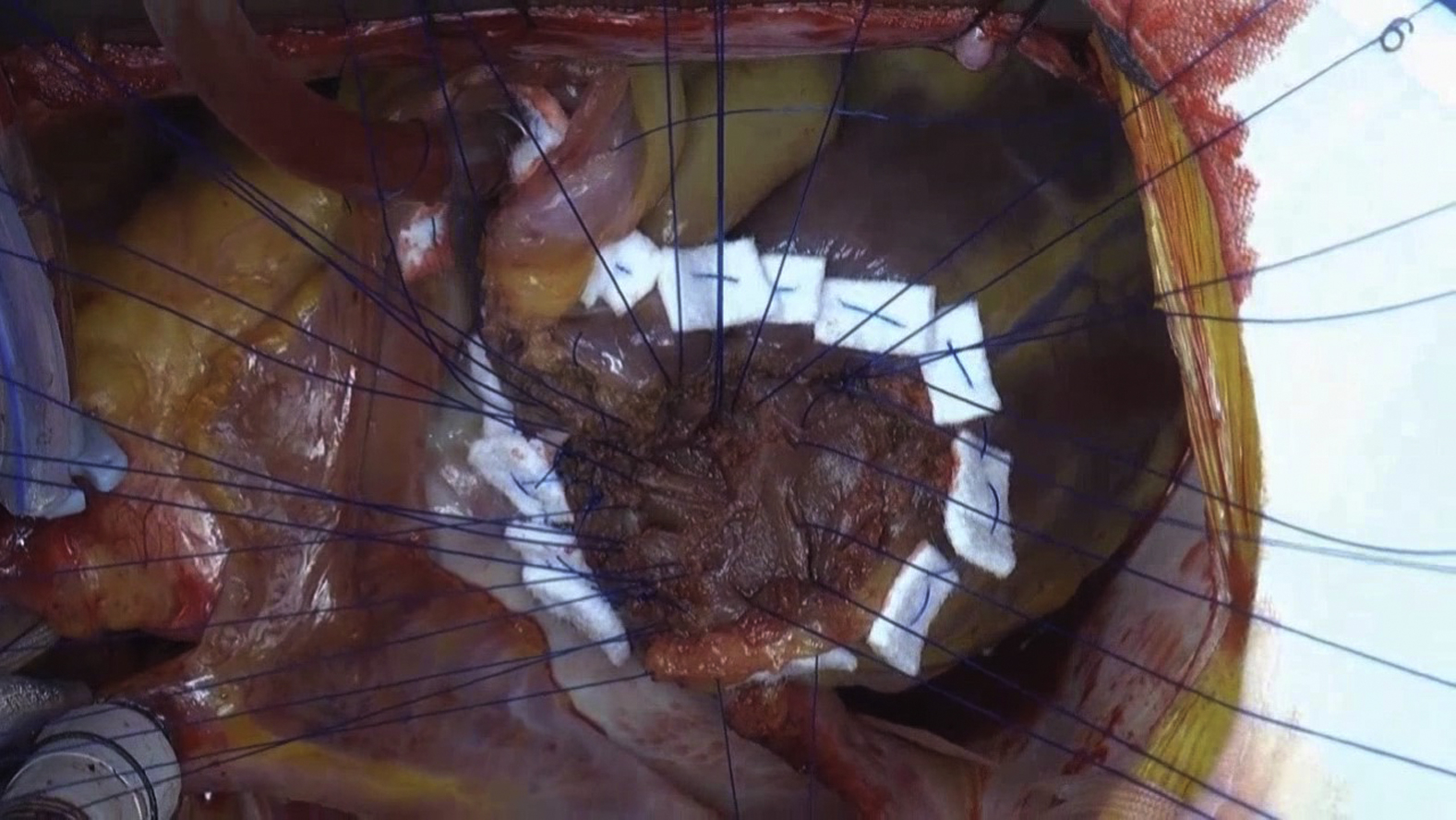

Supplement: Video 1 — The video presents a brief introduction of the patient including preoperative imaging evaluation of the cardiac tumor, surgical procedure, and postoperative course. Video available at: https://www.jtcvs.org/article/S2666-2507(21)00756-2/fulltext. [file fx2.jpg]
